# Differences in the moderating role of supervisors’ and subordinates’ cognition on distributive justice in the relationship between psychological contract and organizational identification

**DOI:** 10.3389/fpsyg.2022.1054940

**Published:** 2022-12-01

**Authors:** Yan Shen

**Affiliations:** School of Health, Shandong University of Traditional Chinese Medicine, Jinan, China

**Keywords:** social exchange theory, psychological contract, organizational identification, distributive justice, cognitive differences

## Abstract

**Introduction:**

In the process of social exchange between employees and organizations, psychological contract, organizational identification, and cognition on distributive justice are closely related and have a common psychological basis, that is, the reciprocity of exchange. The question of how a sense of fairness can affect employees’ psychology and behavior has attracted the attention of scholars and managers.

**Methods:**

The predictive role of psychological contract on organizational identification and the moderating role of supervisors’ and subordinates’ cognition on distributive justice in it were investigated. A paired sample of 133 supervisors and 437 direct reports collected from private service-based SMEs was analyzed through structural equation modeling.

**Results:**

(1) relational psychological contract had a positive predictive effect on organizational identification and transactional psychological contract had a negative predictive effect on organizational identification; (2) subordinates’ cognition on distributive justice played a moderating role in the prediction of psychological contract to organizational identification, and supervisor’s cognition on distributive justice on subordinate’s psychological contract and organizational identification did not reach a significant level.

**Discussion:**

This indicates that there was a significant difference between supervisors’ and subordinates’ cognition on distributive justice.

## Introduction

Social stability depends on a certain degree of fairness, and if the gap between rich and poor is too wide, society is likely to fall apart (Ryu et al., 2021). However, in the actual distribution of benefits, in the face of the distribution results, there are always members who complain about the unfair distribution and thus denigrate the organization or even quit or turnover, while there are always members who quietly accept and identify with the organization regardless of the distribution. In order to reduce employees’ complaints, some companies adopt a non-transparent salary system, but even so, as long as the distribution of benefits is involved, it is inevitable that some employees will complain about unfairness. The question of how a sense of fairness can affect employees’ psychology and behavior has attracted the attention of scholars.

Social exchange theory provides the conceptual basis for studying work attitudes and behaviors (Settoon et al., 1996; Mathafena and Grobler, 2021). People may be attracted to an organization because they expect to receive satisfactory rewards from the organization and want to be accepted by the organization. In order to achieve this, they must give something to the organization (Cropanzano and Mitchell, 2005). Employees seek balance in their social exchange relationship with the organization (Chen et al., 2019), which leads to a cognition on distributive justice. The cognition on distributive justice is the cognition on fairness that employees obtain based on the ratio of their own inputs to outputs and by comparing them with other employees (Cohen, 1987). Employees’ attitudes or behaviors toward the organization depend on their perceived level of fairness (Scheel et al., 2019). In many cases, the parties to an exchange do not seek “absolute” equality of benefits, but rather a relative equality of input–output ratio. When employees perceive the degree of distributive justice, the behavior toward the organization shows three responses: balance, abandonment or modification (Schalk and Freese, 1997).

Employees have expectations of the organization at the beginning of their employment and form an initial psychological contract. Psychological contract refers to a set of implicit agreements of rights and obligations between an employee and a company that are formed on the basis of mutual perception and recognition of the respective expectations of both parties, not through some obvious form of direct and explicit expression of meaning, but through various psychological implication (Kutaula et al., 2020). Rousseau (1990) classified psychological contract into two categories, transactional and relational, from the perspective of social exchange. Transactional contract lacks the characteristics of long-term commitment, and the subject matter that the parties can exchange for a limited period of time includes financial and unique skills, e.g., employees work overtime or improve their performance in exchange for a quick promotion, high compensation, or performance rewards (Ren et al., 2019). Relational contract focus on long-term social exchange at the social psychological level (Ren et al., 2019). and are formed based on mutual trust, e.g., employees exchange loyalty for long-term security or career development. When the transactional type accounts for a larger proportion in the psychological contract, the relational type accounts for a smaller proportion, and vice versa. This pattern has evolved and adapted over time, so contract types are not static.

Organizational identification (OI) refers to the degree to which employees are consistent with their organizations in terms of behaviors and perceptions (Ashforth et al., 2008). Organizational identification comes from many aspects, including corporate brand, corporate awareness, organizational culture, psychological contract, distributive justice, and so on (Boğan et al., 2018; Kashyap and Chaudhary, 2019; Walker, 2021). Among them, psychological contract and distributive justice are psychological variables.

In the process of social exchange between employees and organizations, psychological contract, organizational identification, and cognition on distributive justice are closely related and have a common psychological basis, that is, the reciprocity of exchange. The type of psychological contract is largely formed when employees join the company, however, the perceptions of distributive justice is difficult to be predetermined.

It is important to understand what kind of employees are more responsive to distributive justice and what kind of employees agree with the organization regardless of distributive justice. Furthermore, it is important to explore whether there is any difference between employees’ perceptions of distributive justice align with the factual justice. To answer this question, the employees of private service-based Small and Medium-sized Enterprises (Abbreviated as SMEs) and their direct supervisors were used as the study population. The reason is that private service-oriented SMEs have a strong independence in management decisions and are able to adjust their compensation packages in a timely manner according to the employees’ contributions to the company (Cheng and Waldenberger, 2013). At the same time, private service-based SMEs are under great operational pressure, and employees who value immediate benefits may show different organizational identification than those who value long-term development if compensation or development opportunities are not as good as expected. In other words, in such enterprises, the social exchange relationship is more directly and timely, and the psychological and behavioral phenomena of employees are more easily observed. Therefore, it is examined that how organizational identification changes between employees who value immediate benefits and those who value long-term development under different cognition on distributive justice in private service-based SMEs in this study. To further explore whether subordinates’ cognition on distributive justice was consistent with the factual fairness, and whether subordinates’ cognition on distributive justice plays the same role as the factual justice, their direct supervisors were invited to evaluate the fairness of the distribution scheme. Thus, supervisor-subordinate paired sample design was used for comparative analysis.

## Hypothesis

### The relationship between psychological contract and organizational identification

Both psychological contract and organizational identification emphasize that employees are “aligned” with the company. Psychological contract emphasizes the psychological aspect, while the organizational identification is the employee’s psychological and behavioral consistency with the company, including cognitive, emotional, and behavioral, emphasizing the ‘consistency’ that the employee feels with the organization. Therefore, the relationship between psychological contract and organizational identification is consistent with a logical relationship from psychology to behavior. However, each psychological contract plays a different role for employees (Farnese et al., 2018). If employees take their current job as a springboard and are bent on looking for other job opportunities, they will establish a short-term psychological contract with the organization and are concerned with monetary rewards and need not care about the goals set by the company (Adamovic et al., 2020). As long as they get the expected monetary compensation, employees will be psychologically balanced, otherwise they will be psychologically unbalanced. The more employees value short-term monetary rewards, the less they care about organizational goals, organizational culture, and values. In other words, the stronger the transactional psychological contract, the weaker the organizational identification. If employees establish a long-term relationship contract with the organization, based on trust and commitment, they will be consistent with the organization in many aspects of behavior and perception, and their social exchange will include more social and emotional aspects (such as loyalty, support, and career development plans) other than salary. In other words, the stronger the relational psychological contract, the stronger the organizational identification. Therefore, hypothesis 1 is proposed:

*Hypothesis 1a*: Transactional psychological contract negatively predicts organizational identification.

*Hypothesis 1b*: Relational psychological contract positively predicts organizational identification.

### The moderating effect of subordinates’ cognition on distributive justice on the relationship between psychological contract and organizational identification

To employees, being treated fairly or unfairly is an evaluation about how rewards and punishments are distributed among the group (Tiwari and Jha, 2021). Employees with a relational psychological contract, who expect immediate material rewards for their work, will build trust in the organization if they perceive that the distribution is fair, become psychologically attached to the organization, incorporate the organization’s values into their self-definition, and actively integrate with the organization. When perceived inequity in distribution, they will cope with psychological imbalance and reduce their trust in the organization, which may reduce their efforts or change the form of expectations, or make other negative behaviors that are not conducive to the organization (Clercq et al., 2021), thus bringing their relationship with the organization to a new state of equilibrium. Employees with a relational psychological contract expect long-term development opportunities in the organization in return for their work, so they will not be concerned about temporary gains and losses, or even exchange temporary losses for greater rewards in the future, so a temporary cognition on unfair distribution will not immediately reduce their expectations of the organization and will not affect their organizational identification. When employees feel that the distribution is fair, they will increase their trust in the organization and will feel proud of being a part of the organization (Kim et al., 2021), which will further enhance organizational identification. Therefore, hypothesis 2 is proposed.

*Hypothesis 2a*: Subordinates’ cognition on distributive justice weakens the negative predictive effect of transactional psychological contract on organizational identification.

*Hypothesis 2b*: Subordinates’ cognition on distributive justice strengthens the positive predictive effect of relational psychological contract on organizational identification.

### The moderating effect of supervisor’s cognition on distributive justice on the relationship between psychological contract and organizational identification

Social exchange is an interactive behavior, and both parties to the exchange make judgments about the fairness of the exchange. Therefore, in the exchange process, supervisors and subordinates may have different judgments on distribution fairness due to their different positions. There are two levels of distributive justice, one is the fairness of the objective environment itself and the other is the cognition on fairness by employees. Employees may make judgments that are inconsistent with the facts because of their own perceptual biases. Based on the characteristics of perception, a phenomenon may occur when the objective environment itself is fair, and employees feel unfair, that is to say, there is ambiguity between perception and objective facts (Sherf and Morrison, 2020). At this point, it is necessary to examine the first level, namely， whether the objective environment is fair or not. Since the allocation process is carried out by the supervisor, the supervisor should not only consider the fairness of the current allocation, but also take into account the long-term incentive effect of the allocation results on employees, and may give some resources to some employees. Supervisors see the distribution as fair in the long-term interest of the organization, but not all employees think so. Since subordinates with transactional psychological contract place more importance on the fairness of immediate resource allocation, supervisors’ incentives to other subordinates may cause transactional subordinates to be dissatisfied with the allocation results and thus reduce organizational identification. For subordinates with the relational psychological contract, when they find that their supervisors take into account the long-term interests of the organization in their assignments, they may have greater confidence in their sustainable development in the organization, so that their organizational identification can be further improved. Therefore, hypothesis 3 is proposed:

*Hypothesis 3a*: Supervisory cognition on distributive justice reduces the negative predictive effect of transactional psychological contract on organizational identification.

*Hypothesis 3b*: Supervisors’ cognition on distributive justice strengthens the positive predictive effect of relational psychological contract on organizational identification.

## Materials and methods

### Paired sample design

Theoretically, distributive justice as an objective existence and the evaluation of distributive justice by supervisors and subordinates are measures of two sides of the same concept and should have a fairly high correlation. Based on this idea, many scholars have studied distributive justice by collecting data from only one source, the subordinate (Van Dijke et al., 2019; Jayus, 2021). However, some studies have pointed out that supervisors and subordinates often do not see the same thing in the same way. For example, Gerstner and Day (1997) reported that the correlation between perspectives was.29 in a meta-analytic study, Matta et al. (2015) reported a correlation of only.25.

Social exchange in organizations is an interactive act between two parties, and therefore, both parties make judgments about the fairness of the exchange. Since the perceived value of resources varies from person to person and from situation to situation, there may be inconsistencies in the cognition on distributive justice between the two parties in the exchange process. And these differences in perceptions will further drive the changes and development of the exchange relationship. Therefore, it is necessary to simultaneously examine the cognitive differences between subordinates and supervisors as organizational agents from a two-way perspective.

Therefore, this study conducted a paired sample design to collect data from both supervisor and subordinate levels simultaneously to explore the differences in the perceptions of both sides of the exchange relationship and their roles within the organization.

### Sample and collection process

In this study, 59 private SMEs were selected, and 1–3 teams were selected from each enterprise. The supervisor of each team was asked to select 2 ~ 6 subordinates who had worked in the team for more than 6 months. Finally, 133 teams were collected, including 133 supervisors and 493 subordinates.

To protect the privacy of the participants while ensuring the authenticity of the data, supervisors and subordinates of the same team were asked to come to the same office at the same time to fill in the questionnaires. Supervisor’s evaluation was about the fairness of the assignment program to each subordinate, and each subordinate’s evaluation was about his or her own psychological contract, organizational identification, and the cognition on distributive justice. The real names of the participants were not written on the questionnaires, but each subordinate was named A, B, C, D, E, F, and G by the supervisor, and the subordinates use the code to mark their questionnaires. In the process of recovering the questionnaire, it was necessary to ensure that the supervisor’s evaluation of subordinate A corresponds to the self-evaluation questionnaire of subordinate A, and the supervisor’s evaluation of subordinate B corresponds to the questionnaire of subordinate B, and so on. The questionnaires of the supervisor and his subordinates were bound into a volume corresponding to each other and numbered on the spot.

A valid paired questionnaire means that the supervisor questionnaire and one of his/her subordinates’ questionnaires were successfully paired, and both questionnaires meet the retention requirements. To ensure the validity of the data, the unqualified questionnaires were deleted. The deletion criteria were as follows: The first was the questionnaire with more than 10 consecutive items with the same answer checked, the second was the questionnaire with missing data, and the third was the questionnaire with uncertain pairing relationship. One hundred and thirty-three supervisors’ questionnaires were all qualified, and 437 of 493 subordinates’ questionnaires were qualified and successfully matched with the supervisors’ questionnaires. Finally, 437 valid paired questionnaires were obtained (the effective rate was 88.64%; Table 1).

**Table 1:** Sample.

Demographic variable		Supervisors		Subordinates	
		Frequency	Percentage	Frequency	Percentage
Gender	Male	71	53.38	194	44.39
	Female	62	46.62	243	55.61
Age	≤ 25	3	2.26	71	16.25
	26–30	29	21.8	213	48.74
	31–35	41	30.83	71	16.25
	36–40	33	24.81	37	8.47
	41–45	16	12.03	20	4.58
	46–50	9	6.77	19	4.35
	>50	2	1.5	6	1.37
Education	High school (including below)	12	9.02	65	14.87
	Junior college	41	30.83	269	61.56
	Undergraduate	58	43.61	84	19.22
	Master (including above)	22	16.54	19	4.35
Rank	Generally subordinate			371	84.9
	Low-level managers	74	55.63	58	13.27
	Middle-level managers	56	42.11	8	1.83
	High-level managers	3	2.26	0	0
Work Age	[0.5, 1]	0	0	138	31.58
	(1, 3]	12	9.02	200	45.77
	(3, 5]	23	17.29	38	8.7
	(5, 10]	41	30.83	22	5.03
	(10, 15]	31	23.31	20	4.58
	(15, 20]	26	19.55	19	4.35
Department Scale	[2,5]	46	34.59	144	32.95
	[6,10]	55	41.35	190	43.48
	[11,30]	28	21.05	89	20.37
	[31–50]	4	3.01	14	3.2

### Measures

Each scale was scored on a 5-point scale, with “option 1” to “option 5” indicating the lowest to highest level of compliance.

#### Psychological contract

Millward and Hopkins’ (1998) scale was used, which includes two dimensions, transactional contract and relational contract, with a total of 18 questions. Transactional contract is 1–9 questions, for example, “I work only to achieve my short-term work goals,” Cronbach’s α = 0.88. Relational contract is 10–18 questions, for example, “I expect to be promoted through long-term hard work in the company “. In this study, the internal consistency coefficients for transactional and relational subscales were 0.89 and 0.92, respectively.

#### Organizational identification

Mael and Ashforth (1992) scale was used, with 6 questions in total. For example, “The success of the company is my success.” The internal consistency coefficient of this scale in this study was Cronbach’s α = 0.89.

#### Distributive justice

A 5-item scale developed by Moorman et al. (1993) was used, with an example question such as “I feel that the workload assigned to me by my supervisor is fair.” Based on this, the expression was adapted to the supervisor scale, with the example question “The amount of work I assign to this subordinate is fair.” In this study, the internal consistency coefficient of subordinate’s distributive justice scale is. 87, and that of supervisor’s distributive justice scale is. 81.

## Results

### Data of the sample

SPSS 25.0 was used to analyze the demographic variables of the sample data of supervisors and subordinates, respectively (Table 1). Further normality tests showed that the sample data of supervisors and subordinates were in line with the normal distribution in terms of gender, age, education level, position level, years of employment, and the size of the department they worked in (Table 2).

**Table 2:** Normal test of samples distribution.

	Supervisors (df = 133)				Subordinates (df = 437)			
	Kolmogorov–Smirnov^a^		Shapiro–Wilk		Kolmogorov–Smirnov^a^		Shapiro–Wilk	
	statistical information	significance	statistical information	significance	statistical information	significance	statistical information	significance
Gender	0.404	0.000^***^	0.614	0.000^***^	0.37	0.000^***^	0.632	0.000^***^
Age	0.118	0.000^***^	0.941	0.000^***^	0.192	0.000^***^	0.863	0.000^***^
Education	0.262	0.000^***^	0.829	0.000^***^	0.338	0.000^***^	0.802	0.000^***^
Rank	0.357	0.000^***^	0.731	0.000^***^	0.504	0.000^***^	0.432	0.000^***^
Year	0.192	0.000^***^	0.806	0.000^***^	0.315	0.000^***^	0.633	0.000^***^
Department Scale	0.275	0.000^***^	0.653	0.000^***^	0.264	0.000^***^	0.691	0.000^***^

a Lilliefors significant correction. ^***^p < 0.001.

### Reliability and correlation

SPSS 25.0 was used to analyze the reliability of each variable in turn, and the results showed that the Cronbach’s α of each variable (dimension) was greater than 0.8, indicating a good internal consistency for further analysis. Mean, standard deviation, and correlation coefficient are shown in Table 3.

**Table 3:** Descriptive statistics.

	M	SD	α	CR	1	2	3	4	5
1. Transactional Psychological Contract(PC-T)	2.66	0.84	0.89	0.9	**0.8**				
2. Relational Psychological Contract(PC-R)	3.79	0.7	0.92	0.92	–0.21^**^	**0.83**			
3. Organizational Identification(OI)	3.82	0.73	0.89	0.89	–0.39^**^	0.50^**^	**0.86**		
4. Subordinates’ Cognition on Distributive Justice(OJD-S)	3.65	0.71	0.87	0.88	0.12^**^	0.55^**^	0.20^**^	**0.84**	
5. Supervisors’ Cognition on Distributive Justice(OJD-L)	3.93	0.61	0.81	0.81	0.12^**^	0.09^*^	0.06	0.21^**^	**0.76**

*N = 437*. Bold statistics on the diagonal represent the square root of AVE. ^*^p < 0.05, ^**^p < 0.01.

### Determination of variable independence

In many previous studies, psychological contract (PC) was considered as a whole, but in this study, transactional psychological contract (PC-T) and relational psychological contract (PC-R) were regarded as independent variables, and their correlation reached a significant level (*r* = −. 18, *p* < . 00; Table 4). Although the absolute values of the correlation were not particularly high, it was still necessary to combine the two variables PC-T and PC-R into one variable PC by combining them and then comparing them with the measurement model. Using AMOS 25.0 to verify the measurement model, it was found that in the first model (M1), the items of two structural variables (PC-T and PC-R) were loaded on two different but related potential factors. In the other model (M2), the items of two structural variables (PC-T and PC-R) were forced into the same potential factor—psychological contract (PC). The results of confirmatory factor analysis showed that M1 was better than M2 (Table 4). This shows that transactional psychological contract (PC-T) and relational psychological contract (PC-R) were more suitable to be studied separately as two different variables.

**Table 4:** Fit indexes of competition models.

Model	χ^2^	χ^2^/DF	GFI	AGFI	NFI	CFI	RMSEA	SRMR
Criterion	The smaller the better	≤5.0	≥0.80	≥0.80	≥0.90	≥0.90	≤0.08	≤0.08
M1	159.93	4.70	0.93	0.88	0.95	0.96	0.08	0.06
M2	243.71	9.03	0.89	0.81	0.84	0.85	0.07	0.14
M3	445.99	3.14	0.90	0.86	0.90	0.93	0.07	0.06
M4	252.19	4.00	0.91	0.87	0.92	0.94	0.08	0.07

M1-The hypothetical model composed of PC-T and PC-R. M2-M1’s alternative model composed of PC. M3-The hypothetical model including five variables, PC-T, PC-R, OJD-S, OJD-L and OI. M4-The hypothetical model including three variables, PC-T, PC-R and OI.

### Validity

Confirmatory factor analysis was used to test the fitness indicators of the hypothetical measurement model M3 (including five variables, PC-T, PC-R, OJD-S, OJD-L, OI), and the results were within the acceptable range (Table 4).

Composite Reliability (CR) and Average Variance Extracted (AVE) were used to evaluate aggregate validity (Fornell and Larcker, 1981) and $\sqrt{AVE}$ was used to evaluate differential validity. Fornell and Larcker (1981) suggested that it was acceptable if the CR value was greater than 0.7 and the AVE value was greater than 0.5, while the square root of the AVE should be greater than the correlation coefficient with the other factors. As shown in Table 1, CR and √AVE were both higher than the recommended benchmark, which indicated that aggregate validity and differential validity were sufficient for this study.

### Confirmatory factor analysis

**Figure 1:**
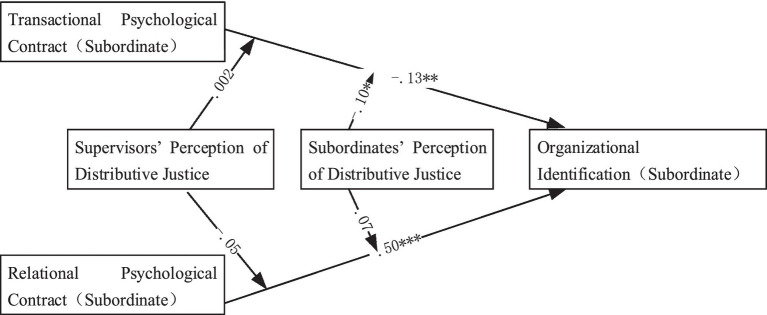
Results of the hypothetical model.

Confirmatory factor analysis was used to test the fitness index of the hypothetical measurement model M4 (including three variables, PC-T, PC-R, and OI), and the results showed good fitness (Table 5). Coefficients from PC-T to OI (*β* = −. 13, *p* < . 01) and PC-R to OI (*β* =. 50, *p* < . 001) reached significant level and were in the same direction as expected (Figure 1), which supported H1a and H1b.

**Table 5:** Moderating effect of OJD-S to the relationship between PC-T and OI.

Variables	Dependent variable: Organizational identification (OI)		
	M1	M2	M3
Gender	0.01	−0.02	−0.02
Age	0.16^*^	0.14^*^	0.13^*^
Education	0.05	0.05	0.05
Rank	0.18^***^	0.14^**^	0.13^**^
Year	−0.08	−0.05	−0.04
Department Scale	0	0.02	0.01
Transactional psychological contract (PC-T)		−0.39^***^	−0.39^***^
Subordinates’ cognition on distributive justice (OJD-S)		0.26^***^	0.23^***^
PC-T × OJD-S			−0.10*
Adj *R^2^*	0.05	0.24	0.25
*ΔR^2^*	0.06	0.19	0.01
F	4.56^***^	56.33^***^	5.05^*^

*N* = 437. *p < 0.05. **p < 0.01. ***p < 0.001; Values in the table were standardized regression coefficients.

### Moderating effect

#### Analysis of the effect of moderating of subordinates’ cognition to distributive justice

Regression analysis results (Table 5) showed that OJD-S moderated the relationship between PC-T and OI (*β* = −0.10, *p* = −0.02). H2a was supported.

**Figure 2:**
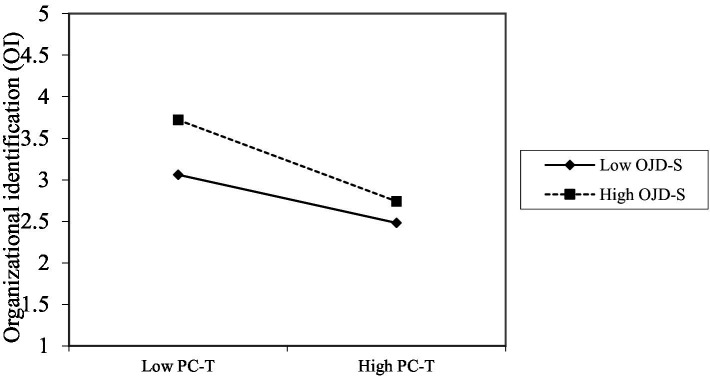
Simple slopes of the direct effect of PC-T to OI at high and low levers of OJD-S.

In order to interpret significant interactions, simple slope analyses and the two-way standardized worksheet by Dawson (2014) were performed (Figure 2). The change of the relationship between transactional psychological contract (PC-T) and organizational identification (OI) was described under the different level of subordinates’ cognition on distributive justice (OJD-S). Subordinates’ cognition on distributive justice (OJD-S) did not change the linear direction of transactional psychological contract (PC-T) to OI, and only changed the degree of the influence. The higher the OJD-S was, the weaker the negative influence of transactional psychological contract (PC-T) to organizational identification (OI) was.

As shown in Table 6, the moderating effect of subordinate’s cognition on distributive justice (OJD-S) to the relationship between PC-R and OI did not reach a significant level (*β* = 0.07, *p* = 0.11). H2a was not supported.

**Table 6:** Moderating effect of OJD-S to the relationship between PC-R and OI.

Variables	Dependent variable: Organizational identification (OI)		
	M4	M5	M6
Gender	0.01	0.01	−0.01
Age	0.16^*^	0.12^*^	0.11^*^
Education	0.05	0.09	0.08
Rank	0.18^***^	0.08	0.09
Year	−0.08	0	0
Department Scale	0	0.01	−0.01
Relational psychological contract (PC-R)		0.52^***^	0.51^***^
Subordinates’ cognition on distributive justice (OJD-S)		−0.06	−0.05
PC-R × OJD-S			0.07
Adj *R^2^*	0.05	0.28	0.28
*ΔR^2^*	0.06	0.23	0
F	4.56^***^	68.12^***^	2.56

N = 437. *p < 0.05, ***p < 0.001; Values in the table were standardized regression coefficients.

For subordinates of relational psychological contract, even if the organization’s decision had a negative impact on individuals, they want to establish a good long-term relationship with the organization, not limited to immediate and short-term interests, and hope to obtain long-term and lasting benefits through exchange (Jones et al., 2018) or other guarantees that will meet their long-term development needs (Gerpott et al., 2019). Therefore, distributive justice did not immediately affect the relationship between psychological contract and organizational identification.

#### Analysis of the effect of moderating of supervisors’ cognition to distributive justice

As shown in Table 7, moderating effect of the supervisor’s cognition on distributive justice (OJD-L) to the relationship between PC-T and OI did not reach a significant level (*β* = 0.002, *p* = 0.97). H3a was not supported.

**Table 7:** The moderating effect of OJD-L to the relationship between PC-T and OI.

Variables	Dependent variable: Organizational identification (OI)		
	M7	M8	M9
Gender	0.01	0.01	−0.01
Age	0.16^*^	0.12^*^	0.12^*^
Education	0.05	0.03	0.03
Rank	0.18^***^	0.18^***^	0.18^***^
Year	−0.08	−0.09	−0.09
Department Scale	0	−0.02	−0.02
Transactional psychological contract (PC-T)		−0.39^***^	−0.39^***^
Supervisors’ cognition on distributive justice (OJD-L)		0.12^**^	0.13^**^
PC-T × OJD-L			0.002
Adj *R^2^*	0.04	0.19	0.19
*ΔR^2^*	0.06	0.15	0
F	4.56^***^	40.63^***^	0

*N* = 437. *p < 0.05, ***p < 0.001; Values in the table were standardized regression coefficients.

As shown in Table 8, moderating effect of supervisor’s cognition on distributive justice (OJD-L) to the relationship between PC-T and OI did not reach a significant level (*β* = −0.05, *p* = 0.25), and therefore, H3b was not supported. That is, supervisors’ cognition on distributive justice cannot directly influence subordinates’ attitudes and behaviors.

**Table 8:** Moderating effect of OJD-L to the relationship between PC-R and OI.

Variables	Dependent variable: Organizational identification (OI)		
	M10	M11	M12
Gender	0.01	0.01	0.01
Age	0.16^*^	0.13^*^	0.13^*^
Education	0.05	0.10^*^	0.11^*^
Rank	0.18^***^	0.09	0.08
Year	−0.08	0.01	0.02
Department Scale	0	0.01	0.01
Department Scale		0.48^***^	0.49^***^
Relational psychological contract (PC-R)		0.04	0.03
Supervisors’ cognition on distributive justice (OJD-L)			−0.05
Adj *R^2^*	0.05	0.27	0.27
*ΔR^2^*	0.06	0.22	0
F	4.56^***^	68.12^***^	2.56

*N* = 437. *p < 0.05, ***p < 0.001; Values in the table were standardized regression coefficients.

#### Differences in the moderating effect of supervisor’s cognition on distributive justice and subordinate’s cognition to distributive justice

The paired sample t-test results showed that (Table 9), there was a significant difference between the cognition on distributive justice of supervisors and that of subordinates (*t* = 7.12, *p* < . 001).

**Table 9:** Paired sample t-test.

		Mean	SD	S.E	95% confidence interval		t	Freedom	Sig.(two-tailed)
					Lower limit	Upper limit			
Pair	DJ-L – DJ-S	0.29	0.84	0.04	0.21	0.36	7.12	436	0

## Discussion and conclusion

### Discussion

First, different types of psychological contract had different effects to organizational identification. The stronger the transactional psychological contract, the worse the organizational identification. If employees establish a transactional psychological contract with the organization, their social exchange with the organization is mainly salary, and they were less concerned about the organization’s development philosophy and social honor. The stronger the relational psychological contract, the higher the organizational identification. If employees had established a relational psychological contract with the organization, their social exchange with the organization will contain more social emotional content beyond salary, so employees will be consistent with the organization in many aspects of behavior and perception. Most of the existing literatures had studied psychological contract as a whole, believing that there is a positive correlation between psychological contract and organizational identification, but there was no consensus on the causal relationship between the two. Some believe that psychological contract is an independent variable. Some considered psychological contract as an independent variable (Alcover et al., 2017; Deng et al., 2018), while others consider organizational identification as an independent variable (Hamzagić, 2018; Tufan and Wendt, 2020). At the same time, there had been more literature on the impact of psychological contract fulfillment or breach on employees’ psychological behavior (Danilwan et al., 2020; Lu et al., 2021; Zhang et al., 2021), and less attention had been paid to the impact of various types of psychological contract on employees’ psychological behavior. This study find that different types of psychological contract had different effects to organizational identification, which provided an empirical basis for research on psychological contract.

Second, in the relationship between different types of psychological contract and organizational identification, subordinates’ cognition on distributive justice played different moderating effects. With the improvement of subordinates’ cognition on distributive justice, the negative impact of transactional psychological contract to organizational identification will weaken, while the positive impact of relational psychological contract to organizational identification will not change significantly. Many studies had focused on the causal relationship between psychological contract and cognition on fairness, but the findings are inconsistent, with some suggesting that cognition on fairness formed stable psychological contract (Huffman et al., 2022) and others suggesting that psychological contract breach had a negative predictive relationship with cognition on fairness (Chen et al., 2021). This study showed that under the transactional psychological contract, the purpose of work was only to achieve personal short-term goals, employees attach importance to the current benefits. The lower the cognition on distributive justice, the stronger the dissatisfaction with the organization With the improvement of the cognition on distributive justice, complaints about the organization will be reduced, and organizational identification will be enhanced. Under the relational psychological contract, employees view the organization as a community of destiny and are concerned about their long-term development opportunities in the organization. They are willing to work hard for the benefit of the organization and share the hardships with the organization, so whether the current benefit distribution is fair or not was not enough to affect their organization identification. Therefore, in order to establish a harmonious working relationship with employees, the organization needs to pay attention to employees’ cognition on distributive justice. On the one hand, it should avoid the strong dissatisfaction of employees with transactional psychological contract caused by low cognition on distributive justice, so as to avoid causing labor conflicts. On the other hand, it should let those employees who plan to develop in the company for a long time to work more happily.

Third, there was a significant difference between supervisors’ cognition on distributive justice and subordinates’ cognition on distributive justice. The correlation coefficient between supervisors’ cognition on distributive justice and subordinates’ cognition on distributive justice was only 0.21, which showed that supervisors’ cognition on distributive justice and subordinates’ cognition on distributive justice were not consistent in most cases. Further relevant sample t-test results showed that there was a significant difference between supervisors’ cognition on distributive justice and subordinates’ cognition on distributive justice. There were two reasons. One reason was that the positions of supervisors and subordinates are different. When formulating the distribution plan, the supervisor not only concerned with the actual contributions of subordinates, but also with the overall and long-term interests of the organization. Supervisors expect their subordinates to use their own skills and talents to create benefits for the company, rather than hiring people to complete assigned tasks step by step, so supervisors tend to use the allocation mechanism to guide the development direction of subordinates. In the process of transformation and upgrading, companies sometimes had to fire certain employees who had given long and loyal service to the company, which can also make employees had a strong cognition on unfairness. In contrast, subordinates focus on the contrast between individual pays and gains, and the cognition on distributive justice is a subjective experience arising from the social comparison of the relative position of individual gain in the team. Therefore, when supervisors think that the distribution is fair, not all subordinates may feel the same as the supervisor. Another reason was that subordinate’ cognition on distributive justice is related to subordinates’ propensity to self-service attributions. Subordinates who were ahead in the distribution tend to attribute positive results to individuals and believe that the higher distribution income is earned by their own hard work rather than due to the tilt of the distribution system. Subordinates who were lagging behind in the distribution tended to attribute the negative results to the situation, believing that they were treated unfairly. It is evident that there is no sole criterion for fairness in allocation outcomes, and the same distribution result had the same impact on subordinates (Kumasey et al., 2021). Subordinates’ subjective experience of fairness is important. The objective fairness of distribution results can only affect subordinates’ psychology and behavior through their subjective experience. Thus, the moderating effect of supervisors’ cognition on distributive justice is not significant in the relationship between various types of psychological contract to organizational identification.

### Theoretical contributions

Most studies involving organizational identification are based on Social Identity Theory (SIT; Wu et al., 2016; Paolino, 2020). And this study uses Social Exchange Theory (SET) to study organizational identification, which is an extension of the application of the theoretical basis of previous studies.

This study proposes that psychological contract and psychological contract fulfillment are two different variables. Psychological contract is the basis of psychological contract fulfillment, and employees conclude some kinds of psychological contract with the organization from the beginning of their employment, and then it is only after the cognitive evaluation that psychological contract fulfillment or breach occurs. Psychological contract is the employee’s inner expectation about how the organization treats him or her, and there are either strong or weak, clear or vague. If psychological contract is weak and vague, employees will not view the exchange relationship from the perspective of psychological contract fulfillment; if psychological contract is strong and clear, employees will view the exchange relationship from the perspective of psychological contract fulfillment.

The psychological contract is not unchangeable when employees are new and after a period of time. The initial psychological contract is formed at the time of entry, and the strength and clarity of the psychological contract will change with the development of the exchange relationship. Therefore, for employees, the strength and weakness and clarity and ambiguity of the psychological contract is more important than the fulfillment and violation of the psychological contract.

### Practical implications

From the perspective of social exchange, this study provides some inspiration for the practice of organizational management.

The main body of enterprise competition relies on talent (Ewers et al., 2022), and in the face of the difficult problem of poor stability of the talent team, it is recommended that private SMEs pay more attention to the type of psychological contract of employees, and help employees improve their organizational identification and enhance their cognition on belonging and mission to the enterprise by adjusting the type of psychological contract.

Supervisors are advised to guide employees to properly analyze the results of their assignments. Subordinates’ perceptions of fairness or unfairness come from personal feelings and are often influenced by personal biases. In the process of social comparison, there is a tendency to overestimate one’s own performance and others’ income and underestimate others’ performance and one’s own income, to see an actual reasonable distribution as unfair, and to see otherwise fair differences as unfair (Schonfeld and Chang, 2017). Therefore, supervisors should promptly understand their subordinates’ perception of unfairness and carefully analyze and guide their subordinates to correctly perceive and treat themselves and others.

It is suggested that supervisors should make good use of the leverage function of the distribution system to balance fairness and development. In the process of social comparison, everyone has a psychological need to seek fairness. Once this need is frustrated, the absolute value of the reward will lose its motivational effect even if it is large. Supervisors should break the egalitarian distribution system. Egalitarianism discourages the motivation of employees who contribute more because they are rewarded equally for unequal contributions. Supervisors should also overcome bias and personal feelings and be fair and reasonable in the distribution process to minimize the objective factors that create a sense of unfairness among subordinates.

### Limitations and future directions

Although there are these advantages, there are also some limitations.

The participants in this study are employees of private service-oriented small and medium-sized enterprises, so all the findings of this study were obtained based on such enterprises. It is not known whether they are applicable to large enterprises. Future comparative studies can be conducted in large enterprises.

There may be deviation in the factual fairness of the distribution results identified by the supervisor’s cognition on distributive justice. Although supervisors had a better understanding of their subordinates’ actual contributions to the enterprise, it is still difficult to avoid the cognition on unfairness caused by the differential treatment of insiders and outsiders. In the future, a third party unrelated to interests should be introduced to evaluate the fairness of distribution results, and the difference between the third party’s evaluation of distribution fairness and the cognition on distributive justice of supervisors and subordinates should be tested.

It needs to be clarified that although the influence of organizational identification is considered positive in most cases, there are sometimes negative effects. While improving employee consistency, organizational identification may also lead to a reduction in the objectivity of evaluation and the act of advising (Blader et al., 2017), decrease in innovative behaviors (Madjar et al., 2011; Zhang et al., 2020), and even the appearance of pro-organization unethical behavior (Effelsberg et al., 2014)， which is not conducive to innovation (Madjar et al., 2011), leading to a reduction in organizational performance (Conroy et al., 2017). Therefore, blindly improving employees’ organizational identification is not the ultimate goal of enterprise human resource management. In future research and management practice, it is necessary to fully consider the two sides of the role of organizational identification and its influencing factors.

## Conclusion

First, different types of psychological contract had different effects to organizational identification. Transactional psychological contract had a negative predictive effect to organizational identification, while relational psychological contract had a positive predictive effect to organizational identification.

Second, in the relationship between different types of psychological contract and organizational identification, subordinates’ cognition on distributive justice plays different moderating role. As subordinates’ cognition on distributive justice increases, the negative effect of transactional psychological contract on organizational identification diminishes, and there was no significant change in the positive effect of relational psychological contract to organizational identification.

Third, there was a significant difference between supervisors’ cognition on distributive justice and that of subordinates.

## Data availability statement

The original contributions presented in the study are included in the article/supplementary material; further inquiries can be directed to the corresponding author.

## Author contributions

The author confirms being the sole contributor of this work and has approved it for publication.

## Funding

This work was supported by Social Science Planning Project of Shandong Province Approval No. 22CGLJ39.

## Conflict of interest

The author declares that the research was conducted in the absence of any commercial or financial relationships that could be construed as a potential conflict of interest.

## Publisher’s note

All claims expressed in this article are solely those of the authors and do not necessarily represent those of their affiliated organizations, or those of the publisher, the editors and the reviewers. Any product that may be evaluated in this article, or claim that may be made by its manufacturer, is not guaranteed or endorsed by the publisher.
